# Complete mitochondrial genome of the Oriental Hornet, *Vespa orientalis* F. (Hymenoptera: Vespidae)

**DOI:** 10.1080/23802359.2017.1292480

**Published:** 2017-03-01

**Authors:** Nizar Jamal Haddad, Kosai Al-Nakeeb, Bent Petersen, Love Dalén, Nikolaj Blom, Thomas Sicheritz-Pontén

**Affiliations:** a Bee Research Department, National Center for Agricultural Research and Extension, Amman, Jordan;; b Department of Bio and Health Informatics, Technical University of Denmark, Lyngby, Denmark;; c Department of Biodiversity Informatics and Genetics, Swedish Museum of Natural History, Stockholm, Sweden

**Keywords:** Oriental hornet, *Vespa orientalis* F, mitochondrial genome

## Abstract

The Oriental Hornet (*Vespa orientalis*) is a social insect belonging to the Vespiade family (Wasps, Hornets, Yellowjackets), genus *Vespa* (true Hornets). The oriental hornet is a scavenger and an agricultural pest, especially to bee farmers, but is also recently described as a harvester of solar energy. Here, we report the mitochondrial genome sequence of the Oriental Hornet, *Vespa orientalis F*., which may play a vital role in understanding this wasp biology, light trapping and generation of electricity. The mitochondrial genome of this hornet is 16,099 bp in length, containing 13 protein-coding genes, 21 transfer RNA genes, and 2 ribosomal RNA genes. The overall base composition of the heavy-strand is 40.3% A, 5.9% C, 13.2% G, and 40.6% T, the percentages of A and T being higher than that of G and C. The mitochondrial genome of the Oriental Hornet, *Vespa orientalis F*. represents the first mitogenome of a solar energy harvesting insect.

The Oriental Hornet, Vespa orientalis F., is distributed throughout the Levant region, Southern Europe, Northeast Africa, and Southwestern Asia including India (Bodenheimer 1951; Haddad Nizar Fuchs et al. 2005). It is known to prey on various insect species, but shows a marked preference for honeybees and is thus considered to be an apicultural pest (Haddad et al. 2007). The hornet causes damage by destroying bee hives and by reducing or even inhibiting the flights of bees (Blum 1956), which results in considerable loss for beekeepers and reduced pollination of crops. It has also become a serious pest on different fruit trees (grape, date palm, pear, peach, citrus, etc.) either by directly destroying the fruits or by the indirect damage coursed by saprophytic fungi. In addition, hornet stings can cause medical problems where individuals react differently to being stung: some are scarcely affected while others suffer considerable pain and swelling (Bodenheimer 1951; Haddad et al. 2007). In recent years, it was discovered that pigments in the hornet’s yellow tissues trap light, while its brown tissues generate electricity. Exactly how the hornets use this electricity is still not entirely understood, but the harvested energy might be used in physical activities, like flight and in temperature regulation (Kaplan 2010; Plotkin et al. 2010). In 2012 we collected and sequenced the genome of an Oriental hornet at the Maru field station in Amman, Jordan (32°36′22.9″N, 35°54′04.9″E). The specimen was disposed during sequencing. We assembled the genome and extracted the mitochondrial sequence from the assembly and annotated it using the MITOS webserver. The mitochondrial sequence reported here adds a new genetic resource for the family Vespadae and will contribute to the understanding of the evolution of sun light harvesting social insects.

The mitochondrial genome of *V. orientalis* is 16,099 bp in length and contains 21 tRNA genes, 13 protein-coding genes, 2 rRNA genes, and 1 non-coding control region (D-loop). The small and large rRNA subunits are 763 and 1378 bp in length, respectively. The overall base composition of the heavy-strand is 40.3% A, 5.9% C, 13.2% G, and 40.6% T. The Cytochrome B gene was split into two parts during annotation. This could be due to internal duplications in that gene; however, the total length corresponds to mitochondrial Cytochrome B genes in similar organisms. We did not observe any overlaps between genes on the same strand. The longest intergenic gap was between the 12s RNA and the gene coding for tRNA-Tyr. The intergenic gap is defined in Figure 1 as the difference between the start and end positions between two adjacent genes. The sequence is available in NCBI Genbank under accession KY563657.

**Figure 1.**
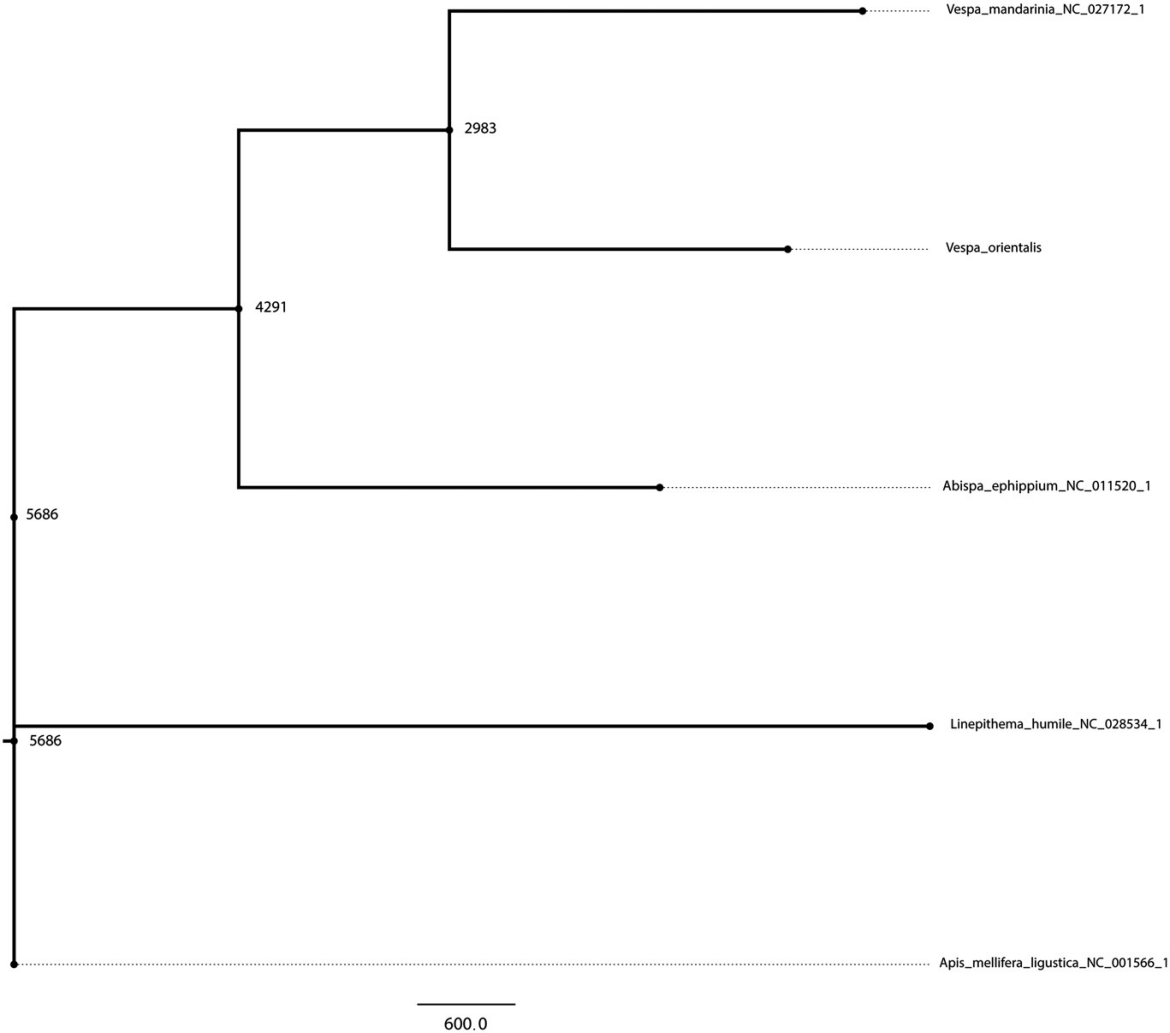
Phylogenetic tree of the Oriental hornet with the Giant Asian hornet (*Vespa mandarinia*), Australian hornet (*Abispa ephippium*), Argentine ant (*Linepithema humile*) and the common honey bee (*Apis mellifera ligustica*) as the outgroup. The tree was created using the parsimony criterion on the ungapped sequences of a multiple sequence alignment.
